# Mindfulness and exercise-related health beliefs: associations with perceived stress dimensions among university students

**DOI:** 10.3389/fpsyg.2026.1853780

**Published:** 2026-09-24

**Authors:** Turan Sezan, Murat Şahbudak, Canan Sarıyar Sezan, Mustafa Enes Işıkgöz

**Affiliations:** 1 Faculty of Sports Sciences, Mardin Artuklu University, Mardin, Türkiye; 2 Faculty of Sports Sciences, Batman University, Batman, Türkiye; 3 Vocational School of Social Sciences, Batman University, Batman, Türkiye; 4 Department of Rectorate, Sakarya University, Sakarya, Türkiye

**Keywords:** health beliefs, mindfulness, perceived stress, protective factors, university students

## Abstract

**Background:**

University students frequently experience stress, appraisals that may negatively relate to health beliefs and motivation for physical activity. Dispositional mindfulness has been identified as a potential psychological resource that may be associated with more adaptive health cognitions under stressful conditions.

**Purpose:**

This cross-sectional study examined associations among the cognitive appraisal dimensions of perceived stress, mindfulness (stress-related inefficacy and helplessness), dispositional mindfulness, and exercise-related health beliefs in university students, testing whether mindfulness is indirectly associated with the relationship. Because the data are cross-sectional, mediation here refers strictly to statistical associations and does not establish temporal or causal relationships or evaluate actual exercise behavior.

**Methods:**

A sample of 1,269 Turkish university students completed validated questionnaires assessing perceived stress appraisal dimensions (stress-related inefficacy and helplessness), dispositional mindfulness, and exercise-related health beliefs based on the health belief model. Structural equation modeling was used to evaluate direct relationships and indirect statistical associations among the latent variables.

**Results:**

Both perceived stress dimensions were negatively associated with mindfulness and exercise-related health beliefs, while mindfulness was positively associated with exercise-related health beliefs. The model accounted for 67% of the variance in exercise-related health beliefs and 45% of the variance in mindfulness. Mindfulness showed significant indirect associations linking both perceived stress appraisal dimensions with exercise-related health beliefs.

**Conclusion:**

These findings indicate that dispositional mindfulness is associated with more positive exercise-related health cognitions in the context of perceived stress appraisals; however, causal or protective effects cannot be inferred. Longitudinal and experimental studies are needed to determine whether mindfulness practices causally influence stress appraisals, exercise cognitions, or actual physical activity participation.

## 1 Introduction

University students face a well-documented range of academic, financial, and social chronic stressors, including academic demands, financial pressures, and social transitions, that consistently undermine their psychological and physical well-being (Duffy et al., 2019; Pascoe et al., 2020). Although regular physical activity is widely recognized as a critical protective resource against these stress-related harms (Herbert et al., 2020), the decision to engage in exercise is not merely a matter of intention; it is fundamentally shaped by cognitive-motivational processes. The Health Belief Model (HBM) offers a useful framework for understanding these processes, positing that health behaviors are driven by specific beliefs about susceptibility, severity, benefits, barriers, and self-efficacy (Alyafei and Easton-Carr, 2024; Ullah et al., 2024). From a stress-appraisal perspective, stress is not defined solely by external demands but also by how individuals cognitively evaluate their ability to manage those demands (Lazarus, 1966, 1999). For students under stress, these exercise-related health beliefs are likely influenced by how they appraise stressful demands – specifically, whether they perceive themselves as capable of coping (stress-related self-efficacy) or as overwhelmed by demands (helplessness) (Khodaveisi et al., 2021; Schönfeld et al., 2016).

In this context, dispositional mindfulness—defined as nonjudgmental awareness of present-moment experience (Kabat-Zinn, 1982, 2003)—emerges as a conceptually distinct psychological resource. Unlike dispositional optimism or resilience, which primarily reflect positive outcome expectancies or general adaptive capacities, mindfulness specifically concerns how individuals relate to their present-moment experiences, particularly stressful ones. The rationale for integrating mindfulness with the HBM and stress-appraisal theory lies in its capacity to support adaptive coping by enhancing emotion regulation and attentional control (Bostock et al., 2019; Petrova et al., 2021). By fostering a decentered, nonreactive stance, mindfulness may disrupt automatic cognitive appraisal processes—such as helplessness-related rumination or inefficacy-related avoidance—that shape health beliefs (Bernstein et al., 2019; Sinha, 2025). Thus, mindfulness is theoretically well suited to function not merely as a direct correlate of perceived stress or health beliefs, but as a potential linking mechanism between how students appraise stress and the health beliefs that guide their exercise-related decisions.

Despite these theoretical connections, the literature reveals two interrelated gaps. First, although mindfulness has been examined separately as a moderator of stress and as a direct predictor of health-related cognitions and behavior, no study to date has integrated these roles to test whether mindfulness serves as an indirect pathway linking perceived stress appraisal dimensions specifically to exercise-related health beliefs (Borges et al., 2025; Garland et al., 2011; González-Martín et al., 2023). Second, previous work has largely operationalized stress as a unidimensional construct, thereby neglecting the possibility that its two cognitive appraisal components—perceived self-efficacy and helplessness—may display distinct associations with health beliefs via mindfulness (Bamert et al., 2025; Diotaiuti et al., 2023). Addressing these gaps is important not only for advancing theoretical understanding of the stress–cognition interface but also for identifying specific, modifiable pathways that could inform future intervention efforts.

To address these gaps, the present study tests a novel conceptual model that positions dispositional mindfulness as both a statistical correlate and a potential indirect pathway connecting perceived stress-appraisal components (stress-related self-efficacy and helplessness) to exercise-related health beliefs within the HBM framework. This approach extends prior research by disaggregating perceived stress into its cognitive appraisal dimensions and explicitly evaluating the indirect, rather than merely moderating or direct, role of mindfulness. Importantly, the present study does not assess specific academic stressors, physiological stress responses, or actual exercise behavior; it focuses on perceived stress-appraisal dimensions and exercise-related health beliefs. Accordingly, the present study addresses the following primary research question: To what extent is mindfulness indirectly associated with the relationships between the self-efficacy and helplessness dimensions of perceived stress and exercise-related health beliefs among university students?

## 2 Conceptual and theoretical framework

### 2.1 Perceived stress and health beliefs in university students

Perceived stress is not a monolithic construct. Consistent with transactional stress theory (Lazarus, 1966, 1999) and its operationalization by Schönfeld et al. (2016), perceived stress comprises distinct cognitive appraisaldimensions: stress-related self-efficacy (confidence in managing demands) and helplessness (feeling overwhelmed). These dimensions are thought to have different influences on health-related cognitions and behaviors, yet their frequent conflation in research hinders a nuanced understanding of their distinct impacts. In the present study, these dimensions represent subjective cognitive appraisals of demands and perceived coping capability rather than specific objective academic stressors or behavioral coping strategies.

Among university students, exercise is consistently associated with reduced stress-related harm (Herbert et al., 2020). The Health Belief Model (HBM) provides a relevant framework for understanding the cognitive processes underlying exercise engagement, positing that health behaviors are shaped by specific beliefs: perceived susceptibility, severity, benefits, barriers, and self-efficacy (Anokye et al., 2023; Fang et al., 2025; Friary, 2024). For students experiencing significant stress, the HBM suggests that strong feelings of helplessness may increase perceived barriers to exercise (such as lack of time, energy, or motivation), while reducing perceived benefits and self-efficacy, thereby weakening positive exercise-related health beliefs (Khodaveisi et al., 2021).

Conversely, lower stress-related inefficacy (i.e., greater coping confidence) may strengthen these beliefs by reinforcing an individual’s perceived ability to integrate exercise and overcome obstacles. Recent empirical work supports this differential relationship: Liao et al. (2023) found that self-efficacy and helplessness uniquely predicted physical activity engagement among university students in the post-pandemic context, with self-efficacy facilitating participation and helplessness inhibiting it. Longitudinal research by Wang et al. (2025) further illustrates the dynamic nature of this process, showing that helplessness from academic stress prospectively increased perceptions of exercise barriers and eroded self-efficacy over time, highlighting a pathway through which chronic stress undermines HBM constructs.

The protective role of stress-related self-efficacy is increasingly emphasized, with evidence indicating that students with higher coping confidence maintain stronger beliefs in the benefits of exercise and greater resilience in sustaining behavioral intentions despite perceived barriers (Leng et al., 2025; Peng et al., 2025). This suggests that within the HBM framework, self-efficacy may be informed not only by task-specific confidence but also by broader perceived competence in managing life stressors, thereby enhancing the model’s explanatory power. Additionally, research identifies significant contextual moderators in these associations: high levels of perceived social support buffer the negative relationship between stress-induced helplessness and perceived exercise barriers, indicating that fostering supportive campus networks may mitigate the adverse effects of helplessness on key HBM components (Fang et al., 2025; Zhang et al., 2025).

In summary, perceived stress in university students is a multidimensional construct whose distinct cognitive appraisal dimensions—self-efficacy and helplessness—engage in complex and divergent relationships with the core tenets of the HBM. Recent research affirms that helplessness primarily increases perceived barriers and undermines self-efficacy, while lower stress-related inefficacy helps preserve beliefs in the benefits of health-promoting actions and in one’s ability to carry them out. Understanding these differential patterns is essential for developing targeted interventions that move beyond global stress reduction to specifically enhance coping self-efficacy and mitigate helplessness cognitions, thereby supporting the maintenance of positive health beliefs under stress.

### 2.2 Mindfulness as a potential linking process

Among the various psychological resources that may moderate relationships between stress and health beliefs – such as optimism, resilience, and self-control—mindfulness offers a conceptually distinct mechanism. Unlike dispositional optimism or resilience, which primarily reflect positive outcome expectancies or general adaptive capacities, dispositional mindfulness specifically targets how individuals relate to their present-moment experiences, particularly stressful ones (Kabat-Zinn, 2003). This decentered, nonreactive stance is theoretically well suited to disrupt automatic cognitive appraisal processes—such as helplessness-related fusion or inefficacy-related avoidance—that may otherwise shape health beliefs (Bernstein et al., 2019; Sinha, 2025).

Previous research has largely examined mindfulness as either a direct correlate of perceived stress or a moderator of stress–behavior relationships in university samples (Lindsay and Creswell, 2017; Waterhouse and Rajvinder, 2025). However, few studies have explicitly tested whether mindfulness operates as an indirect pathway connecting distinct stress-appraisal dimensions (self-efficacy vs. helplessness) to exercise-related health beliefs. The present study extends this literature by positioning mindfulness as a potential intermediary in these associations, rather than treating it solely as a buffer or a separate predictor. Specifically, we test whether mindfulness is associated with cognitive defusion from helplessness-related rumination and with enhanced attentional flexibility that supports stress-related self-efficacy (Taylor et al., 2022), thereby relating indirectly to HBM-based health beliefs.

### 2.3 Hypotheses of the study

Drawing on a theoretical framework that integrates stress appraisal dimensions, dispositional mindfulness, and the Health Belief Model, we propose that both stress appraisal components (PS_SE and PS_H) will show negative associations with mindfulness and HBE, while mindfulness will show a positive association with HBE. Furthermore, we hypothesize that mindfulness will serve as an indirect pathway through which PS_SE and PS_H relate to HBE. Specifically, as shown in Figure 1, we test the following hypotheses:

**Figure 1:**
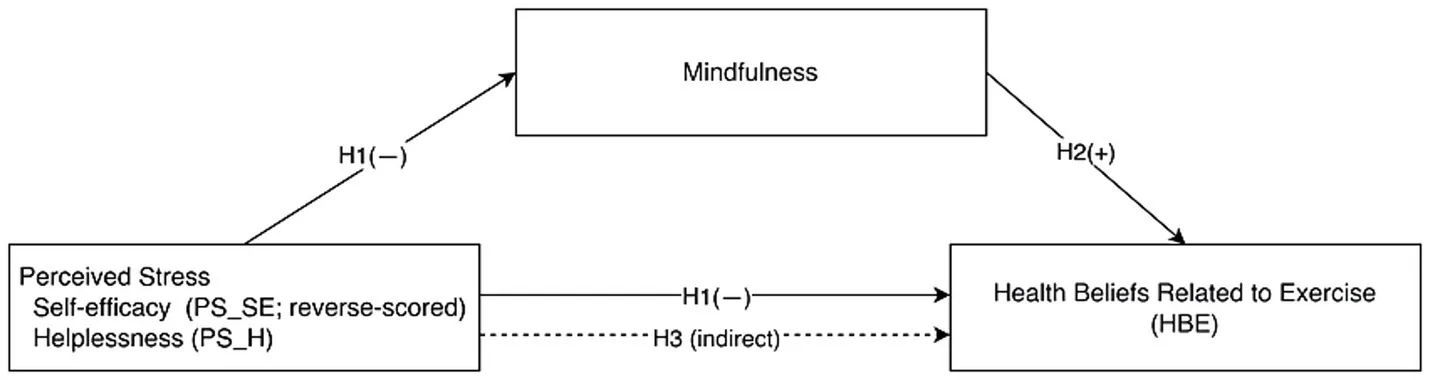
Conceptual research model Note. Solid lines represent direct paths (H1 and H2); H3 represents the indirect paths from Perceived Stress dimensions to HBE through Mindfulness (tested via bootstrapped indirect effects). PS_SE, Perceived Stress–Self-Efficacy (reverse-scored; higher scores indicate greater inefficacy); PS_H, Perceived Stress–Helplessness; HBE, Health beliefs related to exercise.

H1: Perceived stress-related inefficacy (PS_SE) and helplessness (PS_H) will each be negatively associated with both mindfulness and exercise-related health beliefs (HBE).

H2: Mindfulness will be positively associated with exercise-related health beliefs (HBE).

H3: Mindfulness will indirectly link (statistically mediate the associations between) (a) PS_SE and (b) PS_H and HBE.

## 3 Method

### 3.1 Study design

A cross-sectional, correlational survey design was used to examine the relationships among perceived stress appraisal dimensions, dispositional mindfulness, and exercise-related health beliefs. This design is appropriate for assessing these relationships at a single point in time and for testing proposed statistical indirect associations (Levin, 2006). The primary analysis used structural equation modeling (SEM) to evaluate direct relationships and indirect statistical associations. Although SEM can be used to evaluate theoretically specified directional models, the cross-sectional nature of the data precludes causal inference. Therefore, the findings represent statistical associations consistent with an indirect-effects model and do not provide evidence of temporal or causal pathways (Hayes, 2022; Maxwell and Cole, 2007).

### 3.2 Participants

Data were collected via an online survey using convenience and snowball sampling across multiple higher education institutions located in different cities and geographic regions of Türkiye, covering all seven geographic regions (Central Anatolia, Marmara, Aegean, Mediterranean, Black Sea, Eastern Anatolia, and Southeastern Anatolia), during the spring semester of the 2024–2025 academic year. These institutions were selected to capture diversity in geographic location and institutional type, thereby enhancing the representativeness and generalizability of the student sample within the constraints of a non-probability sampling approach. Before participation, all respondents received a detailed information sheet explaining the study’s purpose, voluntary nature, data confidentiality, and their right to withdraw at any time. Written informed consent was obtained from all participants prior to survey completion.

Because this non-probability sampling method limits the representativeness of the findings, this constraint is explicitly noted in the study’s limitations. A total of 1,312 students responded, but data cleaning procedures reduced the final analytic sample to 1,269 participants (age: M = 22.50, SD = 3.17, range = 18–29; 46.6% male, 53.4% female; 73.0% undergraduate, 27.0% graduate; academic field or employment status was not recorded). During screening, 28 participants were excluded for incomplete responses (>10% missing data), and 10 participants were removed for invariant response patterns, defined as providing the same Likert response on ≥90% of the core questionnaire items; this criterion served as a proxy indicator of inattentive or non-differentiated responding. Additionally, five participants were identified as multivariate outliers by computing Mahalanobis distances across the 39 observed questionnaire items. Cases exceeding the critical *χ*^2^ value for df = 39 at *p* < 0.001 [*χ*^2^(39) = 72.055] were excluded. This final sample (*N* = 1,269) exceeds the approximate minimum guideline of 10 observations per measured variable (≈390; Hair et al., 2021), providing adequate power for the planned SEM analyses.

### 3.3 Measurements

#### 3.3.1 Perceived stress scale—short form

The 4-item PSS-2&2 (Schäfer et al., 2023) assesses two cognitive appraisal dimensions: Perceived Stress–Self-Efficacy (PS_SE; 2 items) and Perceived Stress–Helplessness (PS_H; 2 items). Items are rated on a 5-point Likert scale (1 = never to 5 = very often). Following the original scoring procedure, the two PS_SE items are positively worded and were reverse scored prior to analysis; thus, higher PS_SE values indicate greater stress-related inefficacy (i.e., lower coping confidence). The Turkish adaptation Kocapınar and Ekşi (2024) supports the two-factor structure and acceptable reliability. This brief scale assesses cognitive appraisal dimensions of perceived stress rather than specific academic stressors or behavioral coping strategies.

Because each PSS subscale comprises only two items, latent modeling of these subscales requires careful parameterization to avoid identification issues. In the primary confirmatory factor analyses (CFA) and SEM, we used the following recommended procedures to ensure identification and test robustness: (a) latent factors for PS_SE and PS_H were specified with both item loadings freely estimated while the latent factor variance was fixed to 1.0 for identification; (b) parameter estimates and fit were compared with an alternative identification approach (fixing the first item loading to 1.0 and freeing the factor variance). Both parameterizations yielded consistent loading patterns and substantive conclusions.

Additionally, we conducted sensitivity analyses in which PS_SE and PS_H were entered as observed composite scores (item means) in the SEM; the pattern and significance of the main associations and indirect effects were substantively similar. Details of these alternative parameterizations and sensitivity checks, including model fit indices and standardized parameter estimates, are reported in the Results. Finally, reliability and validity indices for the PSS subscales in the current sample showed excellent fit in CFA (*χ*^2^/df = 1.68, CFI = 0.99, TLI = 0.99, RMSEA = 0.02), with standardized loadings of 0.80–0.83; PS_SE: *α* = 0.80, *ω* = 0.80; PS_H: *α* = 0.81, *ω* = 0.81.

#### 3.3.2 Mindful attention awareness scale

The Mindful Attention Awareness Scale (MAAS), developed by Brown and Ryan (2003), is a 15-item self-report measure of dispositional mindfulness (e.g., “I find myself doing things without paying attention”). In the Turkish adaptation (Özyeşil et al., 2011), items were rated on a 6-point scale. The instrument assesses general trait-like mindful attention and does not measure specific meditation practice frequency or intensity. In the present sample, the unidimensional MAAS demonstrated excellent fit in CFA (*χ*^2^/df = 1.94, CFI = 0.99, TLI = 0.99, RMSEA = 0.03), strong internal consistency (*α* = 0.96, *ω* = 0.96), and acceptable convergent validity [Average Variance Extracted (AVE) = 0.62, composite reliability (CR) = 0.96].

#### 3.3.3 Health belief model scale for exercise

The 20-item Health Belief Model Scale for Exercise (HBMS-E), developed by Caz et al. (2023), assesses exercise-related health beliefs across three subscales: Health Improvement Beliefs (HIB; 9 items), Cognitive Health Beliefs (CHB; 6 items), and Sustainability of Exercise (ES; 5 items). Items are rated on a 7-point scale (1 = strongly disagree to 7 = strongly agree). The HBMS-E measures cognitive-motivational beliefs regarding exercise and does not measure actual physical activity behavior (e.g., frequency, duration, or intensity). In the present study, CFA confirmed the three-factor structure (*χ*^2^/df = 3.06, CFI = 0.95, TLI = 0.94, RMSEA = 0.04, SRMR = 0.02), with subscales demonstrating excellent internal consistency and convergent validity (HIB: *α* = 0.98, AVE = 0.83; CHB: *α* = 0.96, AVE = 0.80; ES: *α* = 0.95, AVE = 0.80).

For structural modeling, HBE was operationalized as a second-order latent factor. This decision was guided by (a) theoretical alignment– viewing the HBM subdimensions as facets of a broader cognitive-motivational construct– and (b) strong inter-subscale correlations (*r* = 0.61–0.63), which supported a higher-order representation. To ensure robustness, we performed sensitivity checks by evaluating pathways at both the aggregate (second-order) and subdimension (first-order) levels prior to final estimation; these checks indicated that the second-order specification provided a stable representation of the underlying associations.

Factors across all measures were fixed *a priori* according to the original instruments and their Turkish validations. No *post hoc* modifications, such as introducing residual correlations or removing items based on modification indices, were performed. High CFI and TLI values (approximately 0.99) observed in our CFAs reflect well-specified, theory-driven models rather than overfitting (McNeish et al., 2018). Furthermore, the use of DWLS estimation for ordinal data under conditions of non-normality (Mardia *p* < 0.001) provides robust fit indices for these analyses (Li, 2016, 2021).

To ensure the stability of this specification, specific identification procedures were applied to the 2-item PSS subscales by fixing latent factor variances to 1.0, with results verified against alternative parameterizations. Similarly, the use of a second-order HBE construct was empirically justified by the high inter-subscale correlations (*r* = 0.61–0.63), ensuring that the final structural model remained parsimonious while accurately capturing the common variance of exercise-related health beliefs.

### 3.4 Procedure

This study received ethical approval from the Ethics Committee of Sakarya University of Social Sciences and Humanities (Decision No. E-61923333-050.99-458818, dated March 18, 2025) and was conducted in accordance with the 1964 Helsinki Declaration and its later amendments. Before participation, all respondents received a detailed information sheet explaining the study’s purpose, voluntary nature, data confidentiality, and their right to withdraw at any time. Written informed consent was obtained from all participants through a mandatory electronic checkbox at the beginning of the survey; participants were required to select “I agree to participate” before accessing the questionnaire.

Data were collected via an online survey platform (Google Forms) between April 1 and May 30, 2025. To safeguard participant welfare, the study used only noninvasive, low-burden self-report measures and collected no identifying or sensitive personal information, thereby ensuring complete anonymity. The risk associated with participation was negligible, as the questionnaire addressed common psychological constructs, and no physical or psychological discomfort was anticipated. The perceived benefits—generating evidence to inform student well-being and health promotion strategies—were considered to outweigh this minimal risk. The survey link was distributed through university networks and social media using a snowball sampling approach. The questionnaire consisted of demographic items followed by three scales presented in a fixed order.

### 3.5 Data analysis

All analyses were conducted in Jamovi (v. 2.23.28). Confirmatory factor analyses (CFA) and structural equation modeling (SEM) were estimated using the SEMLj module (lavaan backend). Because the indicators were ordinal, the diagonal weighted least squares (DWLS) estimator was used for all latent variable modeling; DWLS is appropriate for ordinal data and robust to item-level non-normality (Li, 2021). DWLS estimation was based on polychoric correlations among the ordinal indicators.

For descriptive purposes, bivariate associations among observed variables were summarized using Spearman rank correlations, which are less sensitive to non-normal item distributions than Pearson correlations. These are reported separately from the polychoric correlations underlying the SEM/CFA estimation. Model fit was evaluated using conventional indices: chi-square/degrees of freedom ratio (*χ*^2^/df < 3), Comparative Fit Index (CFI > 0.95), Tucker–Lewis Index (TLI > 0.95), RMSEA (≤0.08, with <0.05 indicating close fit), and SRMR (<0.08), consistent with recommended thresholds (Kline, 2023). Convergent validity was assessed via average variance extracted (AVE > 0.50) and significant factor loadings. Discriminant validity was evaluated using Heterotrait–Monotrait Ratio (HTMT) (<0.85) (Henseler et al., 2015), and by confirming that the square root of each construct’s AVE exceeded its inter-construct correlations. Internal consistency was assessed with Cronbach’s alpha (*α*) and McDonald’s omega (*ω*) (Kline, 2023).

To test hypothesized indirect associations, bias-corrected bootstrapping (5,000 resamples) was applied to DWLS-estimated models to derive 95% bias-corrected confidence intervals. All 5,000 bootstrap resamples converged successfully. An indirect association was deemed significant if its 95% CI did not include zero (Hayes, 2022). As a robustness check for common method variance (CMV), we performed Harman's (1976) single-factor test. Because this test is known to have limited sensitivity in detecting CMV, we additionally estimated an alternative model incorporating a latent common method factor, providing a more rigorous evaluation.

## 4 Results

### 4.1 Descriptive statistics and correlations

Descriptive statistics and Spearman rank-order correlation coefficients for study variables are presented in Table 1. Although aggregate scale scores showed acceptable skewness and kurtosis (approximately within ±1.5) (Tabachnick and Fidell, 2021), Spearman correlations were preferred as the primary bivariate summary to conservatively account for item-level ordinality and non-normality. For sensitivity, Pearson correlations were also computed on aggregated scale scores.

**Table 1:** Descriptive statistics and Spearman correlations.

Variables	M	SD	Skewness	Kurtosis	1	2	3	4	5
PS									
1. PS_SE	6.26	1.77	0.17	−0.26	—				
2. PS_H	6.17	1.61	0.26	0.03	0.41^***^	—			
3. Mindfulness	49.13	10.29	0.31	0.81	−0.70^***^	−0.54^***^	—		
HBE									
4. HIB	47.66	10.06	−0.83	0.43	−0.68^***^	−0.55^***^	0.78^***^	—	
5. CHB	32.08	6.36	−0.90	1.06	−0.65^***^	−0.53^***^	0.73^***^	0.78^***^	—
6. ES	26.62	5.29	−1.02	1.13	−0.66^***^	−0.49^***^	0.72^***^	0.75^***^	0.73^***^

*N* = 1,269. Score ranges: PS_SE & PS_H = 2–10; MAAS (Mindful Attention Awareness Scale) = 15–90; HBMS-E subscales — HIB = 9–63, CHB = 6–42, ES = 5–35. Spearman correlations are reported as the primary bivariate summary due to item-level ordinality and non-normality. Sensitivity checks using Pearson correlations on aggregated scale scores yielded substantively similar associations (e.g., Mindfulness–HIB *r* = 0.67; Mindfulness–CHB *r* = 0.59; Mindfulness–ES *r* = 0.57; all *p* < 0.001). Skewness and kurtosis reported in the table refer to aggregated scale scores. ^***^p < 0.001 (two-tailed).

Both perceived stress dimensions were negatively associated with mindfulness (rs = −0.70 for PS_SE, rs = −0.54 for PS_H, *p* < 0.001) and with HBM subscales (rs ranged from −0.49 to −0.68, *p* < 0.001). Mindfulness was strongly and positively correlated with all HBM subscales (rs = 0.72–0.78, *p* < 0.001). Pearson correlations on aggregated scores yielded somewhat lower but substantively similar coefficients for the mindfulness–HBE relationships (mindfulness–HIB *r* = 0.67, mindfulness–CHB *r* = 0.59, mindfulness–ES *r* = 0.57, all *p* < 0.001), consistent with the robustness of the observed association pattern across correlation methods.

Given the magnitude of the mindfulness–HBE associations, we evaluated CMV. Harman’s (1976) single-factor test indicated one factor explaining approximately 51.6% of total variance, which is slightly above the conventional 50% threshold and therefore suggests some shared variance. However, discriminant validity checks (HTMT ratios < 0.85 and √AVE comparisons) supported construct distinctness, and the primary SEM used latent variables with DWLS estimation that explicitly models measurement error. Together, these results suggest that while CMV may partly contribute to the observed associations, it is unlikely to fully account for the strong mindfulness–HBE correlations. We therefore interpret the mindfulness–HBE associations cautiously and note potential conceptual overlap as a limitation.

### 4.2 Measurement model

The theoretical justification for representing exercise-related health beliefs (HBE) as a second-order latent construct is based on the HBM’s conceptualization of beliefs as mutually reinforcing components of a unified cognitive-motivational set (Rosenstock, 1974). Empirically, the second-order model was compared with a first-order model comprising three correlated factors. Both models yielded comparable fit indices (first-order: *χ*^2^/df = 3.06, SRMR = 0.02, RMSEA = 0.04, CFI = 0.95, TLI = 0.94) (Kline, 2023). The change in fit indices between models was negligible (ΔCFI = 0.001; ΔRMSEA = 0.001), well below conventional cutoffs (Cheung and Rensvold, 2002), indicating that the second-order specification did not result in a loss of fit. Given this statistical equivalence and the high standardized loadings from the second-order factor to its subscales (*λ* = 0.82–0.85, *p* < 0.001), the higher-order HBE factor was retained for structural modeling.

The full measurement model– comprising PS_SE, PS_H, mindfulness, and the second-order HBE factor– was tested using the DWLS estimator (Figure 2). This model demonstrated excellent fit (*χ*^2^/df = 1.17, SRMR = 0.03, RMSEA = 0.01, CFI = 0.99, TLI = 0.99). While these near-perfect indices are interpreted cautiously (DWLS in large, well-specified models can yield CFI/TLI values close to 1.0; McNeish et al., 2018), they are consistent with a well-specified measurement structure rather than indicating an absence of measurement error. The constructs showed strong internal consistency (*α*, *ω* > 0.80) and composite reliability (CR range ≈ 0.80–0.95). Convergent validity was supported (AVE range = 0.62–0.68), and discriminant validity was confirmed via HTMT ratios (< 0.85) and √AVE comparisons (Kline, 2023).

**Figure 2:**
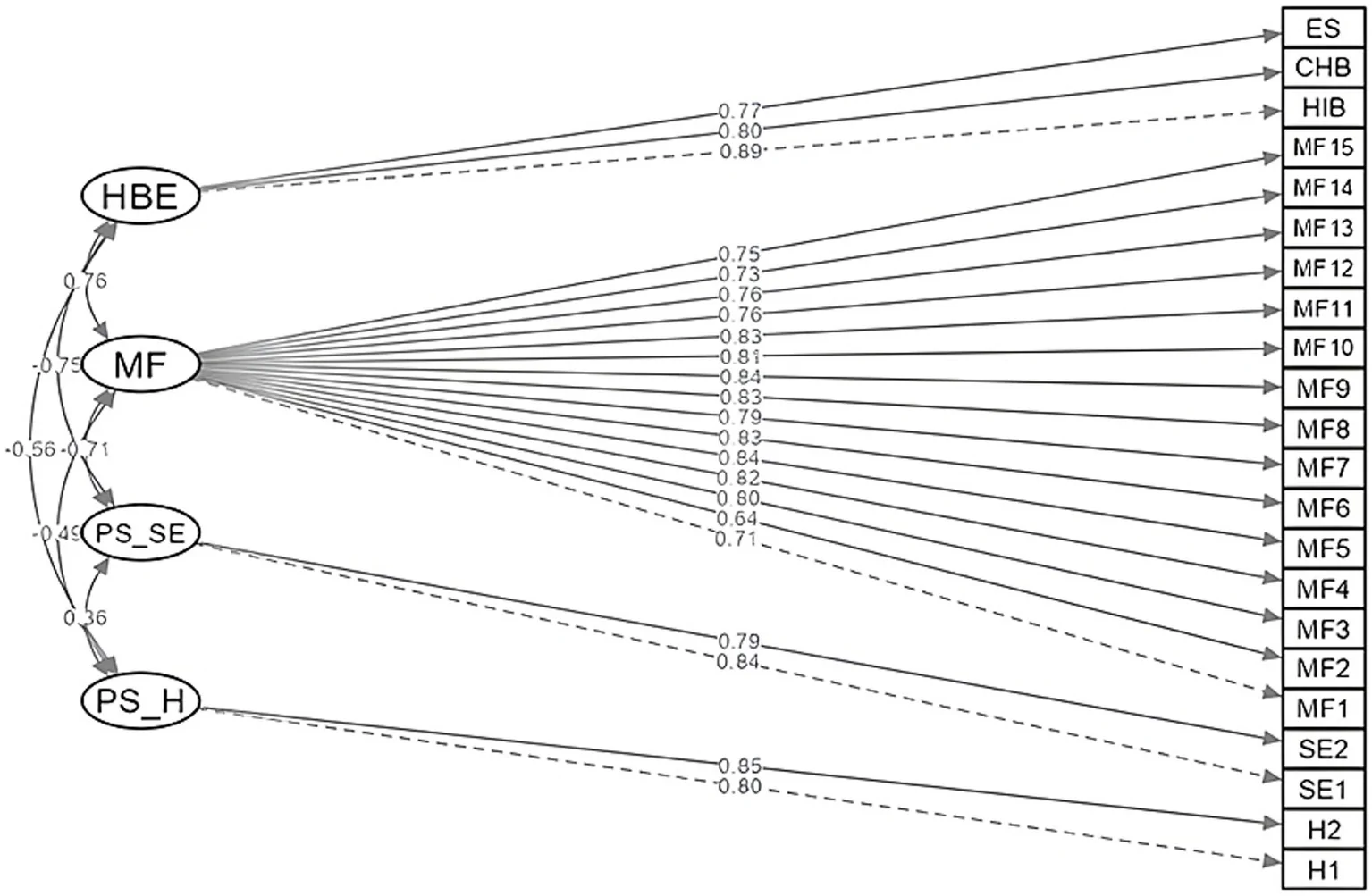
Measurement model. PS_SE, Perceived Stress–Self-Efficacy (reverse-scored; higher scores indicate greater inefficacy); PS_H, Perceived Stress–Helplessness; HBE, Health Beliefs Related to Exercise; MF, Mindfulness.

### 4.3 Structural model and indirect associations

The structural model specified PS_SE and PS_H as predictors, HBE as the outcome, and mindfulness as an intervening variable. Given the cross-sectional nature of the data, these relationships are described strictly as statistical indirect associations rather than as evidence of causal mediation or temporal precedence. The DWLS-estimated structural model demonstrated very good fit (*χ*^2^/df = 1.05, SRMR = 0.01, RMSEA = 0.01, CFI = 0.99, TLI = 0.99) (Kline, 2023). The model accounted for 45% of the variance in mindfulness (*R*^2^ = 0.45) and 67% of the variance in HBE (*R*^2^ = 0.67).

All standardized path coefficients, including direct and indirect effects, are presented in Table 2. Both perceived stress dimensions were negatively associated with HBE [PS_SE → HBE: standardized *β* = −0.33, 95% CI (−0.43, −0.24), *p* < 0.001; PS_H → HBE: *β* = −0.22, 95% CI (−0.28, −0.15), *p* < 0.001]. Mindfulness was positively associated with HBE [Mindfulness → HBE: *β* = 0.45, 95% CI (0.35, 0.55), *p* < 0.001]. PS_SE and PS_H were both negatively associated with mindfulness [PS_SE → Mindfulness: *β* = −0.54, 95% CI (−0.62, −0.47), *p* < 0.001; PS_H → Mindfulness: *β* = −0.27, 95% CI (−0.35, −0.19), *p* < 0.001].

**Table 2:** Standardized direct, indirect, and total effects in the structural model.

Pathway	Direct *β*	95% CI	Indirect *β*	95% CI	Total *β*
PS_SE → HBE	−0.33	[−0.43, −0.24]	−0.24	[−0.30, −0.19]	−0.57
PS_H → HBE	−0.22	[−0.28, −0.15]	−0.12	[−0.16, −0.08]	−0.34
Mindfulness → HBE	0.45	[0.35, 0.55]	—	—	0.45
PS_SE → Mindfulness	−0.54	[−0.62, −0.47]	—	—	−0.54
PS_H → Mindfulness	−0.27	[−0.35, −0.19]	—	—	−0.27

PS_SE, Perceived Stress–Self-Efficacy (reverse-scored); PS_H, Perceived Stress–Helplessness; HBE, Health Beliefs related to Exercise. Total *β* values are the algebraic sum of Direct and Indirect *β* coefficients. Confidence intervals for total effects are not presented but can be derived from the individual components. All effects are standardized (*β*), estimated using DWLS with bias-corrected bootstrapping (5,000 resamples). Model *R*^2^: Mindfulness = 0.45; HBE = 0.67.

Using bias-corrected bootstrapping (5,000 resamples), the indirect association of PS_SE with HBE via mindfulness was significant [*β* = −0.24, 95% CI (−0.30, −0.19), *p* < 0.001], as was the indirect association of PS_H with HBE via mindfulness [*β* = −0.12, 95% CI (−0.16, −0.08), *p* < 0.001]. Including mindfulness reduced but did not fully eliminate the direct associations from stress to HBE, consistent with partial indirect associations.

The large explained variance for HBE (*R*^2^ = 0.67) underscores the strength of the modeled relationships but also warrants caution. Because all measures are self-report and were collected concurrently, CMV and conceptual overlap between mindfulness and exercise-related cognitions may partly inflate estimates. To address this, we conducted multiple checks: (a) discriminant validity tests (HTMT < 0.85 and √AVE > inter-construct correlations) supported construct distinctness; (b) Harman’s single-factor diagnostic (see descriptive statistics) indicated some shared variance (~51.6%) but not a single dominant source; and (c) latent variable SEM with DWLS models measurement error, which reduces observed-score inflation. These checks reduce but do not eliminate concerns about CMV; accordingly, we interpret the large R^2^ for HBE cautiously and discuss these limitations in the Discussion.

Alternative plausible directional specifications (e.g., Mindfulness → PS → HBE; HBE → Mindfulness) were evaluated but did not outperform the hypothesized model in terms of fit or interpretability and did not materially change substantive conclusions. To address identification concerns for the two-item PSS subscales, we re-estimated the structural model using observed composite scores for PS_SE and PS_H; these composite-score models yielded highly consistent estimates (e.g., PS_SE → Mindfulness *β* ≈ −0.56; PS_H → Mindfulness *β* ≈ −0.28; indirect effects PS_SE → HBE ≈ −0.26; PS_H → HBE ≈ −0.13), which supports the robustness of the observed pattern of associations.

## 5 Discussion

This study examined the associations among perceived stress appraisal dimensions, mindfulness, and exercise-related health beliefs in university students, focusing on whether mindfulness functions as an indirect pathway linking stress appraisals to health cognitions. The findings present a nuanced picture: both stress-appraisal dimensions (self-efficacy and helplessness) were negatively associated with exercise-related health beliefs, whereas mindfulness was positively associated with these beliefs and served as a significant indirect pathway. However, the most theoretically novel finding is the asymmetry between the two stress dimensions: the indirect association through mindfulness was considerably stronger for stress-related inefficacy than for helplessness. This pattern suggests that mindfulness may be more closely tied to students’ perceived coping capacity than to their feelings of being overwhelmed—a distinction that has important implications for both theory and intervention.

Supporting our integrated model, both stress-related inefficacy (reverse-scored PS_SE) and helplessness (PS_H) were negatively associated with mindfulness and with exercise-related health beliefs (H1). Students with lower coping confidence and greater helplessness reported less favorable beliefs about the benefits and feasibility of exercise. These findings align with previous research showing that elevated stress undermines motivation for physical activity (Alkhawaldeh et al., 2023; Caz and Yazıcı, 2024; Kandola et al., 2019; Pérez-Jorge et al., 2025; Schuch et al., 2020) and are consistent with the allostatic load perspective, which posits that chronic stress depletes the self-regulatory resources needed for health-promoting behaviors (Ketheesan et al., 2020; McEwen, 1998). At the same time, mindfulness was positively associated with exercise-related health beliefs (H2), supporting the attention-awareness model (Brown and Ryan, 2003) and frameworks emphasizing interoceptive awareness and emotion regulation (Hölzel et al., 2011; Lindsay and Creswell, 2017). Students with higher mindfulness reported greater confidence in the health-enhancing benefits and sustainability of physical activity, suggesting that mindful awareness may help frame exercise as self-care rather than as a burden (Chen et al., 2023; Malik et al., 2024; Williams et al., 2024).

Critically, mindfulness significantly mediated the associations of both stress dimensions with health beliefs (H3). However, the indirect effect was notably stronger for stress-related inefficacy than for helplessness. This asymmetry is theoretically meaningful and constitutes the study’s core contribution. According to Social Cognitive Theory (Bandura, 1997), self-efficacy is a central determinant of health behavior because it directly influences whether individuals believe they can overcome obstacles. Our findings suggest that mindfulness may be particularly relevant for preserving this efficacy belief under stress, thereby indirectly supporting more positive health beliefs (Liang et al., 2024; Lordanić and Tucak Junaković, 2022; Padhy et al., 2022; Wang and Wang, 2025; Wang et al., 2025). In contrast, helplessness—characterized by rumination and perceived loss of control—may be less amenable to mindfulness-related processes, perhaps because it reflects a more global, trait-like orientation toward stressors that is less malleable through dispositional mindfulness alone (Kar and Kar, 2025; Peñacoba-Puente et al., 2025). This differential pattern has not previously been documented in the literature and extends prior work by disaggregating stress into its cognitive components and testing their distinct associations with health beliefs via mindfulness.

Beyond statistical significance, the magnitude of the observed associations also warrants consideration. The standardized indirect effects were meaningful (*β* = −0.24 for stress-related inefficacy and *β* = −0.24 for helplessness), which is typical for studies examining complex psychological processes in nonclinical samples (Funder and Ozer, 2019). Although these effect sizes may not appear large in absolute terms, they are comparable to those reported in meta-analyses of mindfulness and health outcomes (Khoury et al., 2015) and are sufficient to support the theoretical case for mindfulness as a meaningful correlate of health beliefs. From a practical standpoint, these associations suggest that interventions aimed at enhancing mindfulness could yield detectable improvements in exercise-related health beliefs, particularly among students with low stress-related self-efficacy. However, the partial nature of the indirect effects indicates that other resources—such as social support, coping skills, and institutional factors – also play critical roles, and mindfulness training alone is unlikely to be a panacea.

The weaker associations involving helplessness (compared with self-efficacy) require careful interpretation. We consider two plausible explanations. Our preferred interpretation is that the self-efficacy dimension captures a more malleable, state-like cognitive appraisal that is sensitive to mindfulness-related processes, whereas helplessness may reflect a more stable, global orientation toward stressors that is less readily influenced by dispositional mindfulness alone (Schönfeld et al., 2016). This interpretation aligns with research showing that self-efficacy is more responsive to skills-based interventions than helplessness-related cognitions (Bandura, 1997). A secondary, methodological explanation is that the brief helplessness subscale (two items) may have limited the captured variance compared with the self-efficacy subscale (Jiang et al., 2024; Lazarus, 1966, 1999), potentially attenuating the observed associations. We favor the conceptual interpretation but acknowledge that measurement constraints may also play a role.

Given the cross-sectional, self-report design, common method variance (CMV) is a legitimate concern. Harman’s single-factor test indicated that a single factor accounted for approximately 51.6% of the total variance—a value at or above the conventional 50% threshold often used to flag potential CMV concerns (Podsakoff et al., 2003). To evaluate this more rigorously, we estimated an alternative model that incorporated a latent common method factor. In this model, the key path coefficients (e.g., stress → mindfulness, mindfulness → HBE) remained statistically significant and were reduced by only 4–8% in magnitude, suggesting that the substantive associations are not substantially inflated by CMV. However, because the shared variance approached the diagnostic threshold, we acknowledge that CMV may have modestly influenced the magnitude of some estimates. Thus, while we are confident that the reported associations reflect genuine psychological relationships, the effect sizes should be interpreted with appropriate caution and would benefit from replication using multi-method designs (e.g., objective physical activity measures or physiological stress markers).

### 5.1 Implications and future directions

The differential pattern observed for self-efficacy versus helplessness offers a promising direction for future research. If replicated in longitudinal designs, it would suggest that university health promotion programs could benefit from tailoring mindfulness-based interventions to students with low stress-related self-efficacy, rather than applying a uniform approach to all stressed students (Chen et al., 2023; Matos Fialho et al., 2025; Yang and Koenigstorfer, 2021). Future studies should also examine whether mindfulness-based training (e.g., MBSR) produces stronger effects on health beliefs and behaviors among students with low efficacy compared to those with high helplessness. Additionally, incorporating objective indicators—such as physical activity monitors or physiological stress markers—would provide a more comprehensive understanding of these relationships and help disentangle the role of self-report biases.

### 5.2 Limitations and future directions

Several limitations warrant consideration. First, the cross-sectional design precludes causal or temporal inferences; the reported indirect effects represent statistical associations and should not be interpreted as evidence of a causal sequence. Future longitudinal research is needed to determine the temporal precedence of changes in mindfulness. Second, our exclusive reliance on self-report measures may introduce CMV. Although our latent CMV factor check indicated that key paths remained robust, shared variance among self-reported scales warrants cautious interpretation.

Third, a primary substantive limitation is that this study examined exercise-related health beliefs rather than actual physical activity behavior. While health beliefs are established cognitive prerequisites for behavioral engagement, positive beliefs do not guarantee regular exercise adherence. Future research should combine cognitive-belief assessments with device-measured physical activity (e.g., accelerometry) or validated behavioral recall logs.

Fourth, the assessment of perceived stress relied on the brief 4-item PSS-2&2. While psychometrically efficient and suitable for large-scale modeling, it captures cognitive appraisal dimensions rather than specific objective academic stressors (e.g., exam loads, course deadlines) or physiological stress responses. Furthermore, detailed student context variables—such as academic discipline, employment status, and living arrangements—were not collected, which may constrain contextual interpretations. Finally, the Turkish university sample comprises digitally engaged individuals recruited through non-probability sampling, which may limit generalizability to other cultural or socioeconomic contexts. Replications in more diverse populations, including longitudinal models that integrate interpersonal factors such as social support, would provide a more comprehensive understanding of these psychological correlates.

## 6 Conclusion

This study demonstrates that dispositional mindfulness is a significant psychological correlate of how university students maintain exercise-related health beliefs under perceived stress appraisals. Our findings indicate that mindfulness is not only positively associated with favorable health cognitions but also serves as a statistical indirect pathway through which perceived stress dimensions relate to exercise-related health beliefs. Specifically, higher levels of mindful awareness appear to be linked to a greater capacity to perceive the benefits and feasibility of exercise during demanding academic periods.

Theoretically, these results support integrative models that position mindfulness as a protective psychological resource for health-related cognition. Practically, the findings suggest that university health promotion initiatives could benefit from incorporating mindfulness-based strategies to help students navigate stress without compromising their health beliefs. However, the efficacy of such programs must be systematically evaluated through experimental research. In conclusion, fostering mindfulness appears to be a promising strategy to support students in managing academic pressures, potentially helping to preserve the psychological foundations necessary for sustained self-care and resilience.

## Data Availability

The datasets presented in this study can be found in online repositories. The names of the repository/repositories and accession number(s) can be found below: Figshare repository: https://figshare.com/s/fb2465a3c2005017ca82?file=56059010.

## References

[ref1] Alkhawaldeh A. Al Omari O. Al Aldawi S. Al Hashmi I. Ann Ballad C. Ibrahim A. . (2023). Stress factors, stress levels, and coping mechanisms among university students. Sci. World J. 2023, 1–9. doi: 10.1155/2023/2026971, 37426577 PMC10325878

[ref2] Alyafei A. Easton-Carr R. (2024). The Health Belief Model of Behavior Change. Treasure Island, FL: StatPearls. Available online at: https://pubmed.ncbi.nlm.nih.gov/39163427/ (Accessed December 20, 2025). 39163427

[ref3] Anokye R. Jackson B. Dimmock J. Dickson J. M. Kennedy M. A. Schultz C. J. . (2023). Impact of vascular screening interventions on perceived threat, efficacy beliefs and behavioural intentions: a systematic narrative review. Health Promot. Int. 38, 1–15. doi: 10.1093/heapro/daad040, 37279474 PMC10243777

[ref4] Bamert M. Schaffter Y. Bally F. O’Connor D. B. Inauen J. (2025). Assessment of stress and its relationship with health behaviour in daily life: a systematic review. Health Psychol. Rev. 19, 906–936. doi: 10.1080/17437199.2025.2534000, 40844372

[ref5] Bandura A. (1997). Self-Efficacy: The Exercise of Control. New York: W.H. Freeman and Company.

[ref6] Bernstein A. Hadash Y. Fresco D. M. (2019). Metacognitive processes model of decentering: emerging methods and insights. Curr. Opin. Psychol. 28, 245–251. doi: 10.1016/j.copsyc.2019.01.019, 30908987 PMC7459352

[ref7] Borges A. P. Rodrigues P. Brochado A. Sousa A. (2025). Mindfulness as a mediating mechanism between health importance and positive mental well-being. Int. J. Pharm. Healthc. Mark. 19, 773–793. doi: 10.1108/IJPHM-07-2024-0069

[ref8] Bostock S. Crosswell A. D. Prather A. A. Steptoe A. (2019). Mindfulness on-the-go: effects of a mindfulness meditation app on work stress and well-being. J. Occup. Health Psychol. 24, 127–138. doi: 10.1037/ocp0000118, 29723001 PMC6215525

[ref9] Brown K. W. Ryan R. M. (2003). The benefits of being present: mindfulness and its role in psychological well-being. J. Pers. Soc. Psychol. 84, 822–848. doi: 10.1037/0022-3514.84.4.822, 12703651

[ref10] Caz Ç. Paktaş Y. Yazıcı Ö. F. (2023). The validity and reliability study of the health belief model scale for exercises. J. Ege Univ. Nurs. Fac. 39, 115–124. doi: 10.53490/egehemsire.1147705

[ref11] Caz Ç. Yazıcı Ö. F. (2024). Examining the relationship between perceived stress and health beliefs towards exercise. Bozok Int. J. Sport Sci. 5, 247–257.

[ref12] Chen S.-H. Chen P.-J. Lee C.-H. Wu Y.-P. Ahorsu D. K. Griffiths M. D. . (2023). Perceived stress mediating the association between mindfulness and resilience among registered nurses. Psychol. Res. Behav. Manag. 16, 3035–3044. doi: 10.2147/PRBM.S412918, 37576445 PMC10417837

[ref13] Cheung G. W. Rensvold R. B. (2002). Evaluating goodness-of-fit indexes for testing measurement invariance. Struct. Equ. Model. 9, 233–255. doi: 10.1207/S15328007SEM0902_5

[ref14] Diotaiuti P. Valente G. Mancone S. Corrado S. Bellizzi F. Falese L. . (2023). Effects of cognitive appraisals on perceived self-efficacy and distress during the COVID-19 lockdown: an empirical analysis based on structural equation modeling. Int. J. Environ. Res. Public Health 20, 1–16. doi: 10.3390/ijerph20075294, 37047910 PMC10094671

[ref15] Duffy A. Saunders K. E. A. Malhi G. S. Patten S. Cipriani A. McNevin S. H. . (2019). Mental health care for university students: a way forward? Lancet Psychiatry 6, 885–887. doi: 10.1016/S2215-0366(19)30275-5, 31324561

[ref16] Fang L. Zhang Q. Zhou N. Chen J. Lou H. (2025). Influencing factors and mechanisms promoting proactive health behavior intention: An integration of the health belief model and the theory of planned behavior. Front. Public Health 13, 1–15. doi: 10.3389/fpubh.2025.1629046, 40709034 PMC12287080

[ref17] Friary V. (2024). “Mindfulness-based cognitive therapy as a tool to manage emotional and psychological distress,” in Transdiagnostic Approaches in Cognitive Behavioral Therapy, ed. Ornelas A. C. (Cham: Springer), 47–64.

[ref18] Funder D. C. Ozer D. J. (2019). Evaluating effect size in psychological research: sense and nonsense. Adv. Methods Pract. Psychol. Sci. 2, 156–168. doi: 10.1177/2515245919847202

[ref19] Garland E. L. Gaylord S. A. Fredrickson B. L. (2011). Positive reappraisal mediates the stress-reductive effects of mindfulness: an upward spiral process. Mindfulness 2, 59–67. doi: 10.1007/s12671-011-0043-8

[ref20] González-Martín A. M. Aibar-Almazán A. Rivas-Campo Y. Castellote-Caballero Y. Carcelén-Fraile M. D. C. (2023). Mindfulness to improve the mental health of university students. A systematic review and meta-analysis. Front. Public Health 11, 1–23. doi: 10.3389/fpubh.2023.1284632, 38111480 PMC10726040

[ref21] Hair J. F. Hult G. T. M. Ringle C. M. Sarstedt M. (2021). A Primer on Partial Least Squares Structural Equation Modeling (PLS-SEM). Thousand Oaks, California: SAGE Publications.

[ref22] Harman H. H. (1976). Modern Factor Analysis. Chicago: University of Chicago Press.

[ref23] Hayes A. F. (2022). Introduction to Mediation, Moderation, and Conditional Process Analysis: A Regression-Based Approach. 3rd Edn. New York: Guilford Publications.

[ref24] Henseler J. Ringle C. M. Sarstedt M. (2015). A new criterion for assessing discriminant validity in variance-based structural equation modeling. J. Acad. Mark. Sci. 43, 115–135. doi: 10.1007/s11747-014-0403-8

[ref25] Herbert C. Meixner F. Wiebking C. Gilg V. (2020). Regular physical activity, short-term exercise, mental health, and well-being among university students: the results of an online and a laboratory study. Front. Psychol. 11, 1–23. doi: 10.3389/fpsyg.2020.00509, 32528333 PMC7264390

[ref26] Hölzel B. K. Carmody J. Vangel M. Congleton C. Yerramsetti S. M. Gard T. . (2011). Mindfulness practice leads to increases in regional brain gray matter density. Psychiatry Res. 191, 36–43. doi: 10.1016/j.pscychresns.2010.08.006, 21071182 PMC3004979

[ref27] Jiang C. Liu T. Lv J. Ding L. Huang J. Zhang Z. (2024). A structural equation model analysis of the relationship between social support and depression in lung cancer patients: the mediating role of learned helplessness and self-efficacy. Curr. Psychol. 43, 31830–31839. doi: 10.1007/s12144-024-06725-8

[ref28] Kabat-Zinn J. (1982). An outpatient program in behavioral medicine for chronic pain patients based on the practice of mindfulness meditation: theoretical considerations and preliminary results. Gen. Hosp. Psychiatry 4, 33–47. doi: 10.1016/0163-8343(82)90026-3, 7042457

[ref29] Kabat-Zinn J. (2003). Mindfulness-based interventions in context: past, present, and future. Clin. Psychol. Sci. Pract. 10, 144–156. doi: 10.1093/clipsy.bpg016

[ref30] Kandola A. Ashdown-Franks G. Hendrikse J. Sabiston C. M. Stubbs B. (2019). Physical activity and depression: towards understanding the antidepressant mechanisms of physical activity. Neurosci. Biobehav. Rev. 107, 525–539. doi: 10.1016/j.neubiorev.2019.09.040, 31586447

[ref31] Kar B. Kar N. (2025). “Theories in mindfulness studies: a narrative review,” in Health and Wellness for all: Navigating Pathways to a Better Life: A Comprehensive Guide, ed. Batra S. (Cham: Springer), 15–33.

[ref32] Ketheesan S. Rinaudo M. Berger M. Wenitong M. Juster R. P. McEwen B. S. . (2020). Stress, allostatic load and mental health in indigenous Australians. Stress 23, 509–518. doi: 10.1080/10253890.2020.1732346, 32070158

[ref33] Khodaveisi M. Azizpour B. Jadidi A. Mohammadi Y. (2021). Education based on the health belief model to improve the level of physical activity. Phys. Act. Nutr. 25, 17–23. doi: 10.20463/pan.2021.0022, 35152620 PMC8843841

[ref34] Khoury B. Sharma M. Rush S. E. Fournier C. (2015). Mindfulness-based stress reduction for healthy individuals: a meta-analysis. J. Psychosom. Res. 78, 519–528. doi: 10.1016/j.jpsychores.2015.03.009, 25818837

[ref35] Kline R. B. (2023). Principles and Practice of Structural Equation Modeling. 5th Edn. New York: Guilford Press.

[ref36] Kocapınar F. Ekşi H. (2024). Turkish adaptation study of the perceived stress scale short form Ankara International Congress on Scientific Research-X 554–563

[ref37] Lazarus R. S. (1966). Psychological Stress and the Coping Process. New York: McGraw-Hill.

[ref38] Lazarus R. S. (1999). Stress and Emotion: A New Synthesis. New York: Springer.

[ref39] Leng H. Xiang X. Li S. (2025). The chain mediation effects of general self-efficacy and psychological resilience between physical activity and academic stress among Chinese adolescents. Front. Psychol. 16, 1–10. doi: 10.3389/fpsyg.2025.1626157, 40823422 PMC12354399

[ref40] Levin K. A. (2006). Study design III: cross-sectional studies. Evid. Based Dent. 7, 24–25. doi: 10.1038/sj.ebd.6400375, 16557257

[ref41] Li C.-H. (2016). Confirmatory factor analysis with ordinal data: comparing robust maximum likelihood and diagonally weighted least squares. Behav. Res. Methods 48, 936–949. doi: 10.3758/s13428-015-0619-7, 26174714

[ref42] Li C.-H. (2021). Statistical estimation of structural equation models with a mixture of continuous and categorical observed variables. Behav. Res. Methods 53, 2191–2213. doi: 10.3758/s13428-021-01547-z 33791955

[ref43] Liang J. Zhang X. Wang Y. (2024). The relationship between mindfulness and suboptimal health status: a chain/serial mediation model. Front. Psychol. 15, 1–8. doi: 10.3389/fpsyg.2024.1354975, 38572201 PMC10987724

[ref44] Liao C. Nong L. Wu Y.-F. Wu Y.-T. Ye J.-H. (2023). The relationships between university students’ physical activity needs, involvement, flow experience and sustainable well-being in the post-pandemic era. Sustainability 15, 8719–8717. doi: 10.3390/su15118719

[ref45] Lindsay E. K. Creswell J. D. (2017). Mechanisms of mindfulness training: monitor and acceptance theory (MAT). Clin. Psychol. Rev. 51, 48–59. doi: 10.1016/j.cpr.2016.10.011, 27835764 PMC5195874

[ref46] Lordanić L. Tucak Junaković I. (2022). Mindfulness and mental health: the mediating role of cognitive emotion regulation. Drus. Istraz. 31, 577–596. doi: 10.5559/di.31.4.01

[ref47] Malik A. Mussawar B. Fatima H. Mufti A. A. (2024). Stress management techniques for students: exploring mindfulness, cognitive-behavioral strategies and resilience. Rev. Appl. Manag. Soc. Sci. 7, 449–466. doi: 10.47067/ramss.v7i4.393

[ref48] Matos Fialho P. M. Wenig V. Heumann E. Müller M. Stock C. Pischke C. R. (2025). Digital public health interventions for the promotion of mental well-being and health behaviors among university students: a rapid review. BMC Public Health 25, 1–15. doi: 10.1186/s12889-025-23669-1, 40681968 PMC12273279

[ref49] Maxwell S. E. Cole D. A. (2007). Bias in cross-sectional analyses of longitudinal mediation. Psychol. Methods 12, 23–44. doi: 10.1037/1082-989X.12.1.23 17402810

[ref50] McEwen B. S. (1998). Stress, adaptation, and disease. Allostasis and allostatic load. Ann. N. Y. Acad. Sci. 840, 33–44. doi: 10.1111/j.1749-6632.1998.tb09546.x 9629234

[ref51] McNeish D. An J. Hancock G. R. (2018). The thorny relation between measurement quality and fit index cutoffs in latent variable models. J. Pers. Assess. 100, 43–52. doi: 10.1080/00223891.2017.1281286, 28631976

[ref52] Özyeşil Z. Arslan C. Kesici Ş. Deniz M. E. (2011). Adaptation of the mindful attention awareness scale into Turkish. Educ. Sci. 36, 224–235. doi: 10.15390/ES.2011.940

[ref53] Padhy M. Hariharan M. Angiel P. R. Kavya C. Monteiro S. R. (2022). Role of mindfulness in well-being: mediating effect of emotion regulation. Int. J. Happiness Dev. 7, 354–366. doi: 10.1504/IJHD.2022.128032

[ref54] Pascoe M. C. Hetrick S. E. Parker A. G. (2020). The impact of stress on students in secondary school and higher education. Int. J. Adolesc. Youth 25, 104–112. doi: 10.1080/02673843.2019.1596823

[ref55] Peñacoba-Puente C. Gil-Almagro F. García-Hedrera F. J. Carmona-Monge F. J. (2025). From anxiety to hopelessness: examining influential psychological processes affecting mental health status of Spanish nurses during the COVID-19 pandemic. Medicina 61, 236–214. doi: 10.3390/medicina61020236, 40005353 PMC11857786

[ref56] Peng B. Chen W. Wang H. Yu T. (2025). How does physical exercise influence self-efficacy in adolescents? A study based on the mediating role of psychological resilience. BMC Psychol. 13, 285–217. doi: 10.1186/s40359-025-02529-y, 40119462 PMC11927186

[ref57] Pérez-Jorge D. Boutaba-Alehyan M. González-Contreras A. I. Pérez-Pérez I. (2025). Examining the effects of academic stress on student well-being in higher education. Humanit. Soc. Sci. Commun. 12, 1–13. doi: 10.1057/s41599-025-04698-y

[ref58] Petrova K. Nevarez M. D. Waldinger R. J. Preacher K. J. Schulz M. S. (2021). Self-distancing and avoidance mediate the links between trait mindfulness and responses to emotional challenges. Mindfulness 12, 947–958. doi: 10.1007/s12671-020-01559-4, 34149956 PMC8210843

[ref59] Podsakoff P. M. MacKenzie S. B. Lee J. Y. Podsakoff N. P. (2003). Common method biases in behavioral research: a critical review of the literature and recommended remedies. J. Appl. Psychol. 88, 879–903. doi: 10.1037/0021-9010.88.5.879, 14516251

[ref60] Rosenstock I. M. (1974). The health belief model and preventive health behavior. Health Educ. Monogr. 2, 354–386. doi: 10.1177/109019817400200405

[ref61] Schäfer S. K. von Boros L. Göritz A. S. Baumann S. Wessa M. Tüscher O. . (2023). The perceived stress scale 2and2: a two-factorial German short version of the perceived stress scale. Front. Psych. 14:1195986. doi: 10.3389/fpsyt.2023.1195986, 37484682 PMC10358735

[ref62] Schönfeld P. Brailovskaia J. Bieda A. Zhang X. C. Margraf J. (2016). The effects of daily stress on positive and negative mental health: mediation through self-efficacy. Int. J. Clin. Health Psychol. 16, 1–10. doi: 10.1016/j.ijchp.2015.08.005, 30487845 PMC6225043

[ref63] Schuch F. B. Bulzing R. A. Meyer J. Vancampfort D. Firth J. Stubbs B. . (2020). Associations of moderate to vigorous physical activity and sedentary behavior with depressive and anxiety symptoms in self-isolating people during the COVID-19 pandemic: a cross-sectional survey in Brazil. Psychiatry Res. 292, 113339–113334. doi: 10.1016/j.psychres.2020.113339, 32745795 PMC7384423

[ref64] Sinha N. K. (2025). Mediating role of resilience in the relationship between mindfulness and mental health. Front. Psychol. 16, 1–11. doi: 10.3389/fpsyg.2025.1570016, 40735185 PMC12305342

[ref65] Tabachnick B. G. Fidell L. S. (2021). Using Multivariate Statistics. 7th Edn. New York: Pearson.

[ref66] Taylor S. B. Kennedy L. A. Lee C. E. Waller E. K. (2022). Common humanity in the classroom: increasing self-compassion and coping self-efficacy through a mindfulness-based intervention. J. Am. Coll. Heal. 70, 142–149. doi: 10.1080/07448481.2020.1728278, 32150524

[ref67] Ullah F. Ragazzoni L. Hubloue I. Barone-Adesi F. Valente M. (2024). The use of the health belief model in the context of heatwaves research: a rapid review. Disaster Med. Public Health Prep. 18:e34. doi: 10.1017/dmp.2024.26, 38384190

[ref68] Wang M. Wang C. (2025). Exploring pathways to academic engagement: a SEM analysis of self-compassion, social support, and stress management among Chinese university students. BMC Psychol. 13:1207. doi: 10.1186/s40359-025-03332-5, 41174809 PMC12577021

[ref69] Wang T. Wu N. Wang S. Liu Y. (2025). The relationship between psychological resilience, perceived social support, acceptance of illness and mindfulness in patients with hepatolenticular degeneration. Sci. Rep. 15, 1622–1613. doi: 10.1038/s41598-025-85956-6, 39794366 PMC11724018

[ref70] Waterhouse P. Rajvinder S. (2025). University students’ coping strategies to manage stress: a scoping review. Educ. Rev. 78, 127–167. doi: 10.1080/00131911.2024.2438888

[ref71] Williams C. D. Moreno O. Hood K. B. Santana A. Johnson K. F. I-Chun Kuo S. . (2024). Longitudinal associations between well-being and academic achievement throughout the COVID-19 pandemic: testing the moderating role of academic stress among first-generation and continuing college students. J. Divers. High. Educ. 18, 100–110. doi: 10.1037/dhe0000548 PMC12396594 40893708

[ref72] Yang Y. Koenigstorfer J. (2021). Determinants of fitness app usage and moderating impacts of education-, motivation-, and gamification-related app features on physical activity intentions: cross-sectional survey study. J. Med. Internet Res. 23:e26063. doi: 10.2196/26063, 34255656 PMC8317040

[ref73] Zhang K. Liu M. Yin Z. Kong N. Gong H. (2025). The effect of exercise motivation on adolescents’ subjective well-being: chain mediation of physical activity and social-emotional competency. BMC Public Health 25, 1–10. doi: 10.1186/s12889-025-22965-0, 40340715 PMC12060426

